# Polyendocrine Abnormalities Are a Common Phenotypic Feature of the m.3243A>G Variant

**DOI:** 10.5812/ijem-165051

**Published:** 2025-07-31

**Authors:** Josef Finsterer

**Affiliations:** 1 Neurological Dpt., Neurology and Neurophysiology Center, Vienna, Austria

**Keywords:** mtDNA, m.3243A>G, MT-TL1, Heteroplasmy, Stroke-Like Episode

*Dear Editor,*

We refer to the article by Wang et al. about a 6-year-old male with mitochondrial encephalopathy, lactic acidosis, and stroke-like episodes (MELAS) syndrome who phenotypically presented with hyperparathyroidism, bilateral basal ganglia calcification, thyroid cysts, seizures, and lactic acidosis (1). The patient carried the m.3243A>G variant and the clinical presentation was interpreted as MELAS, and the patient was appropriately treated with antiepileptic drugs (ASMs), vitamin D, calcium, and a mitochondrial cocktail, which led to a significant improvement (1). Some issues should be discussed.

The first point is that the criteria used to diagnose MELAS in the index patient were not reported. The diagnostic criteria most commonly applied to diagnose MELAS are those by Hirano et al. (2) or those by Yatsuga et al. (3). The MELAS is diagnosed when a stroke-like episode (SLE) occurs at age < 40 years, and when there are normal early development, lactic acidosis or ragged-red fibres, seizures or dementia, headaches, or vomiting (2). The MELAS is diagnosed according to Yatsuga et al. when there is an appropriate phenotype and a causative mutation (3). Which criteria were used for the index patient?

Secondly the heteroplasmy rates in various tissues were not reported (1). Since heteroplasmy is a determinant of the phenotype (1), it would have been crucial to determine it for the prognosis of the outcome and for genetic counselling. It is also recommended to report the mtDNA copy number, haplotype, and whole exome sequencing (WES) results to assess whether any of the nuclear-encoded mitochondrial genes were additionally involved in phenotypic expression.

The third point is that the index patient never experienced a SLE, the phenotypic hallmark of MELAS (1). The SLEs manifest in cerebral imaging as stroke-like lesions (SLLs), a pathognomonic lesion for MELAS characterized by borders that are not confined to a vascular area, T2/FLAIR, DWI, and PWI hyperintensity, OEF hypointensity, hypometabolism on FDG-PET, and a lactate peak in magnetic resonance spectroscopy (MRS) (4). Did the DWI hyperintensity shown in Figure 1 mentioned in Wang et al. (1) meet the criteria for a SLL?

The fourth point is that lactate levels in the cerebrospinal fluid (CSF) are missing (1). Since the patient had elevated serum lactate levels, it is very likely that elevated lactate levels were also present in the CSF. Was MRS ever performed on the patient to determine whether or not there was a double lactate peak? Knowing whether or not lactate is present in the CSF is crucial, as CSF lactate acidosis can trigger seizures.

The fifth point is that we disagree with the caption for Figure 1 mentioned in Wang et al. in the index study (1). Contrary to the description that the patient had a DWI hyperintense lesion in the right parieto-occipital region, we cannot detect any lesion in the parietal region. The lesion shown in Figure 1 mentioned in Wang et al. (1) is located exclusively in the right occipital region.

In summary, MELAS should only be diagnosed if the Hirano or Japanese criteria are met and if the m.3243A>G variant is present in a heteroplasmic state. Polyendocrine involvement is a common feature of MELAS.

## Data Availability

All data are available upon a reasonable request from the corresponding author.

## References

[1] Wang L, Zhou T, Bu X, Pan D, Liu X (2025). Hypoparathyroidism in a Child with MELAS Syndrome: A Case Report of Severe Lactic Acidosis and Symmetrical Bilateral Basal Ganglia Calcification.. Int J Endocrinol Metab..

[2] Hirano M, Ricci E, Koenigsberger MR, Defendini R, Pavlakis SG, DeVivo DC (1992). Melas: an original case and clinical criteria for diagnosis.. Neuromuscul Disord..

[3] Yatsuga S, Povalko N, Nishioka J, Katayama K, Kakimoto N, Matsuishi T (2012). MELAS: a nationwide prospective cohort study of 96 patients in Japan.. Biochim Biophys Acta..

[4] Finsterer J (2023). Characteristics of stroke-like lesions on cerebral imaging.. Ideggyogy Sz..

